# Impact of Head Trauma: How MMA (Mixed Martial Arts) Affects the Sense of Smell, Cognition, and Quality of Life in Fighters

**DOI:** 10.1055/s-0045-1810027

**Published:** 2025-10-16

**Authors:** Guilherme Basso Durães, José Lucas Barbosa da Silva Bortoleto Guerino, Ellen Cristine Duarte Garcia, Richard Louis Voegels, Fábio de Rezende Pinna, Marco Aurélio Fornazieri

**Affiliations:** 1 Department of Clinical Surgery, Universidade Estadual de Londrina (UEL), Londrina, PR, Brazil; 2 GEM, Londrina, PR, Brazil; 3 Department of Otorhinolaryngology, Faculty of Medicine, Universidade de São Paulo, São Paulo, SP, Brazil; 4 Department of Medicine, Pontifical Catholic University of Paraná, Londrina, PR, Brazil; 5 Department of Otorhinolaryngology: Head and Neck Surgery, Smell and Taste Center, Perelman School of Medicine, University of Pennsylvania, Philadelphia, United States

**Keywords:** olfaction disorders, hyposmia, athletic injuries

## Abstract

**Introduction:**

The sense of smell plays an essential role in well-being, influencing relationships, the formation of emotional memories, and protection against toxic substances in food and the environment. Post-traumatic olfactory loss is among the leading causes of olfactory alterations, especially in contact sports like Mixed Martial Arts (MMA), which involve frequent physical trauma, particularly to the head. To date, the relationship between olfactory dysfunction and head trauma in fighters remains underexplored.

**Objective:**

To evaluate possible olfactory, gustatory, and cognitive dysfunctions in MMA fighters. Methods: Fourteen MMA fighters and 14 control participants, matched for sex and age, were recruited. The participants underwent tests to evaluate olfaction (Visual Analog Scale (VAS) and the University of Pennsylvania Smell Identification Test (UPSIT), taste (Modified Global Gustatory Test), cognition (Montreal Cognitive Assessment, MoCA) and quality of life (The 36-Item Short Form Health Survey questionnaire, SF-36).

**Results:**

A statistically significant difference was found in olfactory function between the groups (p = 0.021), with fighters having a lower mean score than the control group (fighters: 28.5 ± 4.4 vs. controls: 31.8 ± 2.4). Additionally, MMA fighters showed cognitive decline and impairment in quality of life aspects. No difference was observed in gustatory function between the groups (p = 0.508).

**Conclusion:**

The results indicate that MMA fighters exhibit impairments in olfactory function, as well as in cognition and quality of life. Further studies with larger sample sizes are needed for a more in-depth analysis of the impact of this sport on athletes' lives.

## Introduction

The sense of smell is fundamental to well-being, playing a crucial role in social interactions, the formation of emotional memories, and protection against toxic substances present in the environment or food. 1 Patients with olfactory dysfunction may experience a significant decline in their quality of life, including a reduced enjoyment of food and difficulty in identifying environmental hazards. Additionally, studies indicate that olfactory loss is associated with higher rates of depression, highlighting its emotional and psychological impact. 2 3

Post-traumatic olfactory loss is one of the main causes of olfactory dysfunction due to the vulnerability of the olfactory bulb, which is located above the cribriform plate in the brain's anterior region. 4 Head trauma can affect brain areas involved in olfaction, such as the orbitofrontal cortex, frontal lobe, and the anteroinferior portion of the temporal lobe. 5 The olfactory epithelium, located in the upper part of the nasal cavity, contains neurons that detect odor molecules and transmit this information to the olfactory bulb. 6 7 From there, the information is relayed to the olfactory cortex and other brain regions, such as the hippocampus and hypothalamus, which are responsible for integrating olfactory perceptions. 7 Traumatic injuries can cause rupture of the olfactory nerve, 8 contusions in the orbitofrontal cortex, 9 or obstructions that impair communication between the olfactory epithelium and the central nervous system. 10

Combat sports, such as Mixed Martial Arts (MMA), expose athletes to frequent head trauma, potentially compromising olfactory function. Studies with former NFL players (National Football League) show that reduced performance in olfactory tests is associated with poorer neuropsychological and neuropsychiatric functioning. 11 However, the relationship between olfactory dysfunction and repetitive head trauma in MMA fighters remains underexplored. Investigating olfactory function in these athletes may provide crucial insights into the sport's impact on olfactory health and quality of life, contributing to the development of protection and treatment strategies.

## Methods

### Study Design and Population

This was an observational, cross-sectional, case-control study. The study included professional MMA athletes over 18 years of age and control group participants who did not practice this or any other sport that could cause head trauma or who had complaints of smell and taste loss. The control group participants were matched for sex and age. Individuals with neurological or psychiatric disorders; those with a history of head and neck cancer; chronic rhinosinusitis; history of stroke; traumatic brain injury from other causes; colds on the day of the test; or those reporting smell loss after COVID-19 were excluded from the study. Participants were invited to take part through announcements on social media. The study was approved by the Research Ethics Committee involving human subjects (approval protocol number: 4.592.321) and all participants signed the informed consent form.

### Olfactory Assessment

The olfactory assessment of participants was conducted using a Visual Analog Scale (VAS) and the University of Pennsylvania Smell Identification Test (UPSIT ^®^ ). The UPSIT ^®^ consists of four booklets, each containing 10 odors, with one odor per page. At the bottom of each page, there is a brown strip with plastic microcapsules containing the odor stimuli. Participants were instructed to scratch this strip with a pencil to release the odor and then select the option that best described the scent. The final score is based on the number of correct answers, classifying olfactory function as normosmia, hyposmia (severe, moderate, or mild), or anosmia. 12

### Gustatory Assessment

The gustatory function was evaluated using a Modified Global Gustatory Test with four flavors: salty, sweet, sour, and bitter. The salty flavor was derived from a 0.31M sodium chloride solution; the sour flavor from a 0.015M citric acid solution; the sweet flavor from a 0.49M sucrose solution; and the bitter flavor from a 0.04M caffeine solution. The solutions were presented in oral spray form. The examiner presented 16 flavors (four of each type) in a predetermined random order. Participants performed the test without knowing the contents of each vial and were required to choose one of the four flavors. The test score was based on the total number of correct responses (0 to 16 points), and gustatory function was classified as normogeusia (13-16), mild hypogeusia (10-12), moderate hypogeusia (7-9), severe hypogeusia (4-6), and ageusia (<4). 13

### Cognitive Assessment

Cognitive function was assessed using the Montreal Cognitive Assessment (MoCA), and the results were compared with standardized norms for the questionnaire. The MoCA is a brief global cognitive scale developed to detect individuals with mild cognitive impairment. 14 Additionally, it is a practical method for identifying subtle cognitive changes, with good internal consistency, reliability, and content validity. 14 The MoCA has been translated and adapted for use in Brazil, with studies suggesting it is a valid and reliable tool for screening mild cognitive impairment in the Brazilian population. 15 16 The test consists of a 30-point assessment administered over 10-15 minutes, with 10 subtests evaluating visuospatial skills, naming, working memory, attention, concentration, calculation, repetition, verbal fluency, abstraction, short-term memory, and orientation to time and place. 14 The test duration is estimated at 20 minutes, with a maximum possible score of 30 points. A score below 26 points indicates mild cognitive impairment, while scores above 26 are considered normal. 14

### Quality of Life Assessment

Quality of life was assessed using “The 36-Item Short Form Health Survey questionnaire (SF-36)”. Data from the fighters were compared with the normative data of the Brazilian population for the 25-34 age group. This is a widely used tool for assessing health-related quality of life, measuring eight domains: physical functioning, role physical, bodily pain, general health, vitality, social functioning, emotional role, and mental health. 17 Each domain is scored from 0 (worst) to 100 (best).

### Statistical Analysis

After assessing the distribution of numerical variables with the Shapiro-Wilk test for normality, appropriate statistical tests were applied. For normally distributed variables, Student's t-test was used to compare means between groups, while the Mann-Whitney U test was applied for non-normally distributed variables. Categorical variables were analyzed using Fisher's exact test to account for small sample sizes or expected frequencies. All tests were two-tailed, and a p-value < 0.05 was considered statistically significant.

## Results

Fourteen fighters who regularly participated in competitive matches and 14 control group participants were recruited. The groups were matched for age and sex. The average age in both groups was 33.2 years (SD ± 8; p = 1), from 22 to 49 years old. All participants were male (p = 1).

**Fig. 1:**
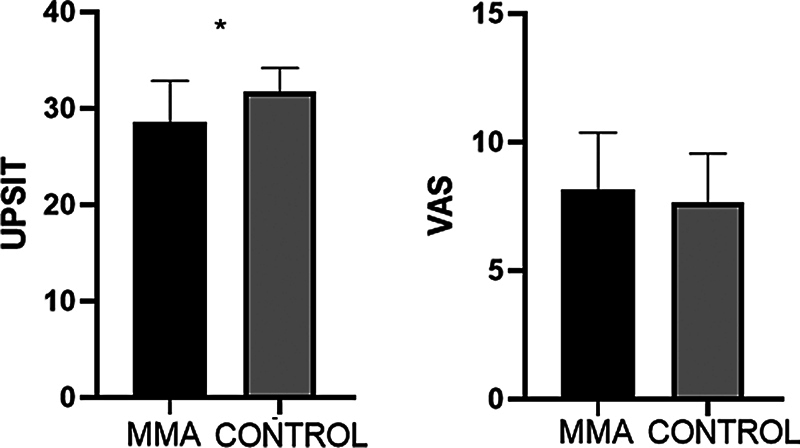
Comparison of mean scores on the University of Pennsylvania Smell Identification Test (UPSIT ^®^ ) and the Visual Analog Scale (VAS) between MMA (Mixed Martial Arts) fighters and the control group. UPSIT ^®^ (University of Pennsylvania Smell Identification Test), MMA (Mixed Martial Arts fighters), VAS (Visual Analog Scale). *Statistically significant value (p = 0.021).

The comparison of mean scores on the olfactory test (UPSIT ^®^ ) between the groups showed a statistically significant difference (p ¼ 0.021), with the fighters' group having a lower average score than the control group (fighters: 28.5 SD: 4.4 vs. control: 31.8 SD: 2.4). This olfactory loss is consistent with mild hyposmia. When comparing the means related to the Visual Analog Scale (VAS) for smell, there was no statistically significant difference between the groups (fighters: 8.1 SD: 2.2 vs. controls: 7.7 SD: 1.9, p ¼ 0.563) ( Fig.1 ).

The correlation between fight time and UPSIT ^®^ was negative, but the value was not statistically significant (p = 0.312, r = -0.356). When comparing the relationship between cognition and UPSIT ^®^ , a positive correlation was found, but the value was not statistically significant (p = 0.06, r = 0.514).

There was no statistically significant difference when comparing the scores obtained by the fighters and the control group regarding taste evaluation (controls: 14.07, SD: 2.06 vs. fighters: 13.29, SD: 2.84, p = 0.508).

**Fig. 2:**
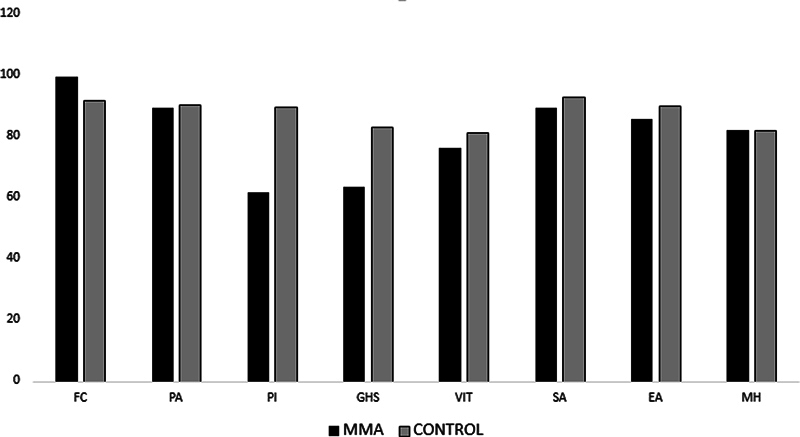
Comparison of the average scores of fighters and the Brazilian normative values for each domain of the quality of life questionnaire (SF-36). FC = Functional capacity; PA = Limitation due to physical aspects; PI= pain; GHS = General Health Status; VIT = Vitality; SA = Social Aspects; EA= Limitation by emotional aspects; MH = Mental Health.

When comparing the 8 scales (functional capacity, limitations due to physical aspects, pain, general health status, vitality, social aspects, limitations due to emotional aspects, and mental health) of the quality of life questionnaire (SF-36), only in the domains “functional capacity” and “mental health” did the fighters' average exceed the Brazilian normative average, as shown in the graph below. In the other domains, there was a decrease in indices ( Fig. 2 ). The cognitive assessment of the fighters, as measured by the MoCA test, was 24, a value below 26, which is considered the cutoff for mild cognitive impairment (MCI) in this test.

## Discussion

The present study demonstrated a statistically significant difference in olfactory loss among MMA fighters compared to a non-fighting control group, consistent with hyposmia. Although the study has a limited sample size, it yields important results since this topic is rarely studied and published in literature. The findings highlight a critical factor to be considered by practitioners of the sport.

A previous study conducted with a population of boxers showed significantly reduced olfactory performance compared to a non-boxing control group. 18 Studies indicate that a single occipital blow can lead to rupture of the olfactory nerve due to shear forces on the cribriform plate. 8 Nasal trauma can induce changes in airflow directed to the olfactory region, caused by mucosal edema, deformities in the nose, nasal skeleton, meatus, and nasal conchae affected by facial blows. 19 Another point to highlight is that several studies have demonstrated that various cranial traumas caused by boxing generate lesions in the olfactory bulb and temporal lobe, confirmed by magnetic resonance imaging, consequently leading to olfactory dysfunction. 20 21 Participants did not report a decline in olfactory function on the visual analog scale, indicating they were unaware of the changes occurring. This may be attributed to the loss being mild, but the delay in recognizing this loss could lead to a worse treatment prognosis.

Furthermore, MMA is associated with cognitive impairment compatible with mild cognitive impairment (MCI) and impacts several domains of quality of life, such as increased limitations due to physical aspects, perception of physical pain in daily life, deterioration of general health status, and vitality, along with impairments in social aspects and emotional limitations.

The taste function of the participants did not appear to be affected by the sport; although the fighters had a lower average than the controls, this difference was not statistically significant. However, with the deterioration of olfaction, there may be a subsequent decline in the perception of flavors by individuals.

Literature lacks studies regarding the consequences of MMA on fighters' lives, including olfactory and gustatory function, quality of life, and cognition. A limitation of our study is the small size of the sample analyzed. Despite this, the present study provides results indicating the impairment of these variables in athletes' lives. A study with a larger number of participants, along with follow-up throughout their sports practice and complementary imaging studies assessing the olfactory bulb, is suggested to better elucidate the mechanisms of these impairments and explore ways to protect and minimize damage to the athletes.

## Conclusion

The results of the study indicate impairment in the olfactory function of MMA fighters, consistent with hyposmia, as well as in cognition and quality of life. No alterations in taste were observed. Future studies with larger samples are needed for a more in-depth analysis of the impacts of this sport on the lives of athletes.
